# On the Anatomical State of the Skin and Subcutaneous Cellular Tissue, in Yellow Fever

**Published:** 1822-02

**Authors:** A. Desmoulins

**Affiliations:** the Faculty of Medicine of Paris, Member of the Natural History Society, &c.


### MORBID PHYSIOLOGY.

**Art. VI.**—***On the Anatomical State of the Skin and subcutaneous Cellular Tissue, in Yellow Fever.*** **By A. Desmoulins, M.D. of the Faculty of Medicine of Paris, Member of the Natural History Society, &c. [Read at the Royal Institute of France, and communicated to the Editor of the London Medical and Physical Journal.]**

AMONG the causes of the existing discrepancy of opinion respecting the nature of yellow-fever, we may fearlessly mention the small number of necrotomic inspections that have been made in cases of that disease; and the little accuracy with which those inspections have been conducted. Every author, who has written on the subject, has mentioned, in a desultory manner, the sanguineous congestion observed in the parenchymatous viscera; the red patches found on the pulmonary and costal pleuræ, as well as on the mucous membrane of the stomach and duodenum; and, lastly, the presence of a brownish, and sometimes sanguinolent, fluid in the bladder. But no conclusive facts have been recorded, except what are due to Dr. Ffirth. This physician has ascertained that the matter ejected in what has been called the black vomit in yellow-fever, does not proceed from the liver, as it was generally supposed; for he has found the same black fluid in the stomach of patients who had been affected, during that disease, by the *vomito prieto*, and in whom the pylorus was observed to be completely obstructed by scirrhus, so that the smallest communication could not be traced between the stomach and duodenum. Now, as in these patients the fever had, throughout, exhibited its ordinary type, no exception can be said to have occurred, to account for the singular fact which they exhibited, and to which I have just alluded. If, therefore, the black matter of the vomit did not proceed from the liver, it must have been the product of vascular exhalation. This unavoidable conclusion becomes an object of demonstration, if, as Dr. Ffirth affirms, the same black matter, ready formed, has been actually met with in the smaller arteries of the stomach. A fact entirely analogous to this has been recently mentioned by Dr. Breschet, in his interesting memoir on Melanosis.* This gentleman has proved, both anatomically and chemically, that the matter of melanosis is formed by the blood only; and he states having found a substance, altogether similar to that of melanosis in the blood-vessels which terminated in the organs affected by that disease. Lastly, in the majority of subjects examined by Dr. Ffirth after death, the state of the liver, as well as the quantity and quality of the bile found in the gall-bladder, were indicative of a diminution, rather than an increase, in the hepatic secretion.

It is easy to perceive to what important inductions we may be led, by these facts, in the treatment of the fever in question.

Authenrieth, and others, have remarked, that the serum has been found of a yellow colour in many diseases, in which there were no symptoms of bilious complication. The skin of elderly persons, in perfect health, has occasionally become yellow. From some observations made by Dr. Breschet, at the Foundling Hospital, on the bodies of infants who had died of icterus, it appears that no traces of increased bile, or of deviation of that fluid from its right course, could be found in them. The yellow colour of the skin has induced superficial observers into error. This phenomenon is the result of peculiar changes taking place in the circulation of the blood. To this I may add, that the icterus is often only partial, and that foundlings are more exposed to it than other infants, owing, probably, to the sudden diminution of temperature which takes place in them, on passing from the uterus into the common atmosphere. M. Dalmas, quoted by Dr. Breschet in his memoir, has had occasion, frequently, to observe the healthy state of the liver and biliary secretion in cases of yellow fever; and he has remarked, that the yellowness of the skin would often commence by large bands over the course of the blood-vessels, instead of appearing first on the conjunctiva, as is the case in common jaundice. I may, also, observe, that there exists a whole race of the human species, in whom the skin is of a decidedly yellow colour; that the race in question is not more subject to biliary affections than any other; that this peculiar colour of the skin is the result of a change effected in the blood contained in the vascular network of the dermoid surface, known under the name of the *rete mucosum* of Malpighi; and, lastly, that certain conditions of the body, whether physiological, such as pregnancy, or pathological, occasion, in the different races of mankind, anomalous discolorations of the skin, either local or general.

* For an account of the memoir of Dr. Breschet, alluded to by the author, we beg to refer our readers to the account we have given of it in the department of MEDICAL BIBLIOGRAPHY for this month.

It results from all these facts, that the yellow colour of the skin may occur independently of any morbid increase in the secretion of the bile, or deviation from its right channels: and it is not improbable that, in the yellow-fever, the discoloration of the skin may, like the black matter vomited in that disease, be the effect of some cause unconnected with the secretion of bile. The observations of Dr. Ffirth prove the correctness of this assertion, as far as it relates to the latter phenomenon; while my own experience leads me to believe that it is correct, also, with regard to the former.

In the month of July, 1815, a man was brought to the Hôtel Dieu at Rouen, with every symptom of fever, having arrived the previous day at Havre, in twenty-two days, from Martinique. He complained of severe head-ach, and soon afterwards became delirious; so much so, indeed, as to require being secured by means of a straight-waistcoat. The most prominent symptoms were—intense redness of the eyes; dimness of sight; hiccup; partial hemorrhage from the nose, mouth, and anus; perspiration, which left yellow stains on the linen; echymosis; and, on the third day, general appearance of jaundice. From this period, the black vomit began, and the patient appeared at times to lose his sight completely: coma ensued, partial convulsions followed, and death happened on the fifth day.

The body was opened five hours after death, while yet preserving a considerable degr.e of warmth. The skin was of a yellow colour, particularly on the cheeks, in the axillæ, in the groins, and, in general, wherever the subcutaneous cellular tissue is most abundant. On making an incision into the skin, a drop or two of blood oozed out; and, on the scalpel penetrating into the cellular tissue, a quantity of gas escaped, with a whistling noise, through the incision: the part, at the same time, became considerably *affaissée*. The laminæ of the cellular tissue presented an extremely fine and injected vascular network, as we observe in the case of inflamed conjunctiva. The colour of the vessels was of a reddish brown. This phenomenon was equally observed in the axillary and inguinal regions, as well as in the parotid glands. The stomach, cœcum, and colon, contained a black and adhesive matter, similar to what had been evacuated during life, by vomiting and by the rectum. The mucous membrane of the larger intestines, in particular, and of the stomach, was of a brownish red, with numerous patches of a deeper colour. The liver exhibited no particular appearance; and the gall-bladder, without being distended, contained a proper quantity of a yellowish-brown healthy bile.

There were, at that time, in the hospital, several patients ill of typhus, sent from the army; in some of whom all the phenomena above described were observed, though in a lesser degree, after death, when the examination had been made before the body had entirely lost its natural heat.

Here, then, we have two anatomical facts, hitherto unnoticed, doubtlessly, because the necroscopic examinations have been made some considerable time after death.—1. The exhalation of gas from the cellular tissue. 2. The injection of the laminæ of that tissue with blood, which naturally does not penetrate thus far, corresponding with a similar injection of the capillary vessels of the skin. This state of the cellular tissue and of the skin, analogous to that which is observed on the irritated surfaces of blistered parts, and the production of gas, seem to indicate a tendency of humours towards the skin, which might not unaptly be assimilated to what has been remarked in hemorrhagic congestions of contiguous mucous membranes: and, if no hemorrhage takes place through the skin, is it not merely on account of its very close texture? The difference in the appearance of the black matter vomited or passed by the rectum, when compared to that of the blood in hemorrhages which take place from the nose, the mouth, or the anus, is not a proof that the former is not itself originally hemorrhagic; since it is well known that the aspect, and probably the composition also, of the blood in hemorrhages, vary according to the surfaces from which they emanate, and in proportion to the degree of irritation of those surfaces.

From these facts, which are peculiarly my own, connected with those that I have quoted from other authors, I think I am warranted in adopting the following conclusions:

1. That, in yellow-fever, there is no morbid increase in the secretion of bile.

2. That the intestinal surface exhale the black matter observed in cases of vomit, as well as in the alvine evacuations.

3. That the yellow colour of the skin is the result of a peculiar change taking place in the vascular net-work of that integument, in which a species of congestion (fluxion) obtains, analogous to that which is observed in the case of hemorrhage of the mucous membranes of the intestines.

4. That the close texture of the skin is the only obstacle to an actual hemorrhage taking place from it.

5. That the yellow colour of the skin, which is, generally, preceded by petechiæ and echymoses, as I have had occasion to observe, is, in fact, only a species of general echymosis.

6. Lastly, that the yellow fever consists in a sanguineous congestion, occurring simultaneously under the skin and in the mucous membranes, particularly of the digestive organs, with different degrees of intensity, and modified by the different degrees of permeability of the membranes themselves with respect to the transmission of blood through them.

